# Dome-shaped macula in children and adolescents

**DOI:** 10.1371/journal.pone.0227292

**Published:** 2020-01-07

**Authors:** Eunhae Shin, Kyung-Ah Park, Sei Yeul Oh

**Affiliations:** Department of Ophthalmology, Samsung Medical Center, Sungkyunkwan University School of Medicine, Seoul, Republic of Korea; University of Warmia, POLAND

## Abstract

**Purpose:**

We sought to evaluate the incidence and characteristics of dome-shaped macula (DSM) in children and adolescents with myopia.

**Methods:**

A retrospective review of medical records was performed to identify subjects who were younger than 19 years with myopia of −3.0 diopters or greater. The results of optical coherence tomography images were analyzed to identify DSM. The height and diameter of the dome were measured. Age, best-corrected visual acuity (BCVA), and refractive error of study participants who exhibited DSM were compared with those of patients who did not.

**Results:**

Among the 1,042 eyes of 615 patients, eight eyes (0.77%) of seven patients had DSM. Six of these eight eyes were not highly myopic (i.e., less than −6.0 diopters of spherical equivalents). Additionally, the mean height and diameter of the identified domes were 146.50 ± 42.33 μm and 4779.75 ± 699.38 μm, respectively. Patients with DSM were significantly older (mean age: 15.88 ± 2.36 years) than patients without it (11.51 ± 4.60 years; p = 0.007). The youngest affected patient was 11 years old. There was no significant difference in refractive errors (p = 0.629) or BCVA (p = 0.314) between the two groups.

**Conclusions:**

Although the incidence in this study was very low, DSM was found even in children and adolescents. In addition, 75% of affected individuals were not highly myopic. These results suggest that inherent traits may be involved in development of DSM.

## Introduction

A dome-shaped macula (DSM) is a distinct entity that is characterized by inward elevation of the macula [1]. Previous optical coherence tomography (OCT) studies have revealed that a thick sclera under the macula is responsible for this elevation [2]. The clinical significance of DSM is that it is associated with a low incidence of foveoschisis and retinal detachment related to macular holes, but also with increased incidence of serous macular detachment [3, 4].

Since DSM is usually found in highly myopic eyes [1, 4, 5], it has been postulated that the origin of DSM is closely associated with anatomical changes secondary to myopia progression or staphyloma [1, 2, 6]. However, the exact origin of DSM has not yet been fully elucidated. The evaluation of DSM in affected subjects may provide useful data on the condition’s origin. However, since previous studies have mainly incorporated data from adults [1–4, 7–11], limited knowledge exists regarding the characteristics of DSM in children or adolescents [10, 12, 13].

The purpose of the present study was to evaluate the incidence and characteristics of DSM in children and adolescents.

## Materials and methods

The institutional review board and ethics committee of Samsung Medical Center in Seoul, Republic of Korea approved this retrospective study. The study was conducted according to the principles expressed in the Declaration of Helsinki.

A retrospective review of medical records was performed to identify patients under 19 years who had visited the pediatric ophthalmology and neuro-ophthalmology clinic of Samsung Medical Center between January 2009 and October 2018. Among them, patients who exhibited ≤−3.0 diopters (D) of spherical equivalent (SE) at the initial visit were included. The following were excluded: 1) patients without available OCT images; 2) patients with a history of vitreoretinal surgery; and 3) patients with a history of macular disease that may affect the morphology of the macula, such as retinal dystrophy or retinal detachment.

At their initial visit, all the patients underwent a comprehensive ophthalmologic examination, including slit-lamp biomicroscopy, fundus examination, and measurement of best-corrected visual acuity (BCVA). In patients under 15 years old, cycloplegic refraction was routinely performed at initial visits to assess refractive error. Cycloplegic refraction was performed approximately 45 minutes after the instillation of 3–5 drops of cyclopentolate 1% and tropicamide 0.5%. In patients older than 15 years, cycloplegic refraction was usually performed at initial visits. In some instances, however, manifest refraction was performed to assess refractive error at the discretion of the doctor. SE was calculated as the sphere power plus half of the cylinder power. Horizontal and vertical OCT cross-hair scans centered at the center of the fovea were performed using the Spectralis^®^ device (Heidelberg Engineering, Heidelberg, Germany).

DSM was defined as the presence of an inward bulge of the retina and sclera on vertical or horizontal OCT. Based on the previously suggested definition [4, 8, 9], a dome height greater than 50 μm was considered to represent DSM.

**Fig 1:**
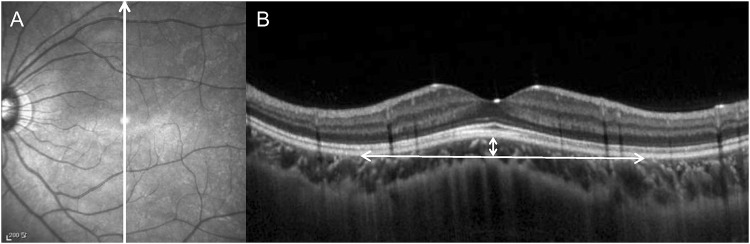
Method for the measurements of the diameter and height of the dome. (A) Infrared image showing the method for the measurements of the diameter and height of the dome. A white arrow indicates an optical coherence tomography scanning line. (B) Optical coherence tomography image showing the method for the measurements of the diameter and height of the dome. The diameter of the dome base was defined as the distance between the borders of the dome (horizontal double-headed arrow). The height of the dome was defined as the vertical distance between the top of the dome and the measurement line for the diameter of the dome (vertical double-headed arrow).

The height and diameter of the dome region were measured using the Heidelberg Eye Explorer software (Heidelberg Engineering) (Fig 1). A horizontal line running parallel to the line connecting the outer surface of the retinal pigment epithelium at the base of the dome was drawn. The distance between the two connecting points was defined as the diameter of the dome. According to the method used in the previous studies [8, 9], the vertical distance between the horizontal line and the anterior surface of the retinal pigment epithelium at the fovea was defined as the height of the dome.

The incidence and characteristics of DSM among the included subjects were identified. In addition, age, sex, SE, and BCVA were compared between patients with and without DSM. In eyes with DSM, the occurrence of Bruch’s membrane defects was also assessed based on OCT images.

Statistical analysis was computed using the Statistical Package for the Social Sciences version 24 software program (IBM Corp., Armonk, NY, USA). Data were presented as means ± standard deviations or numbers (percentages) if applicable. The BCVA values were converted to logarithm of minimal angle of resolution (logMAR) values for statistical analyses. Comparisons between two groups were analyzed using the Mann–Whitney *U* test or Fisher’s exact test. A p-value less than 0.05 was considered significant.

## Results

**Fig 2:**
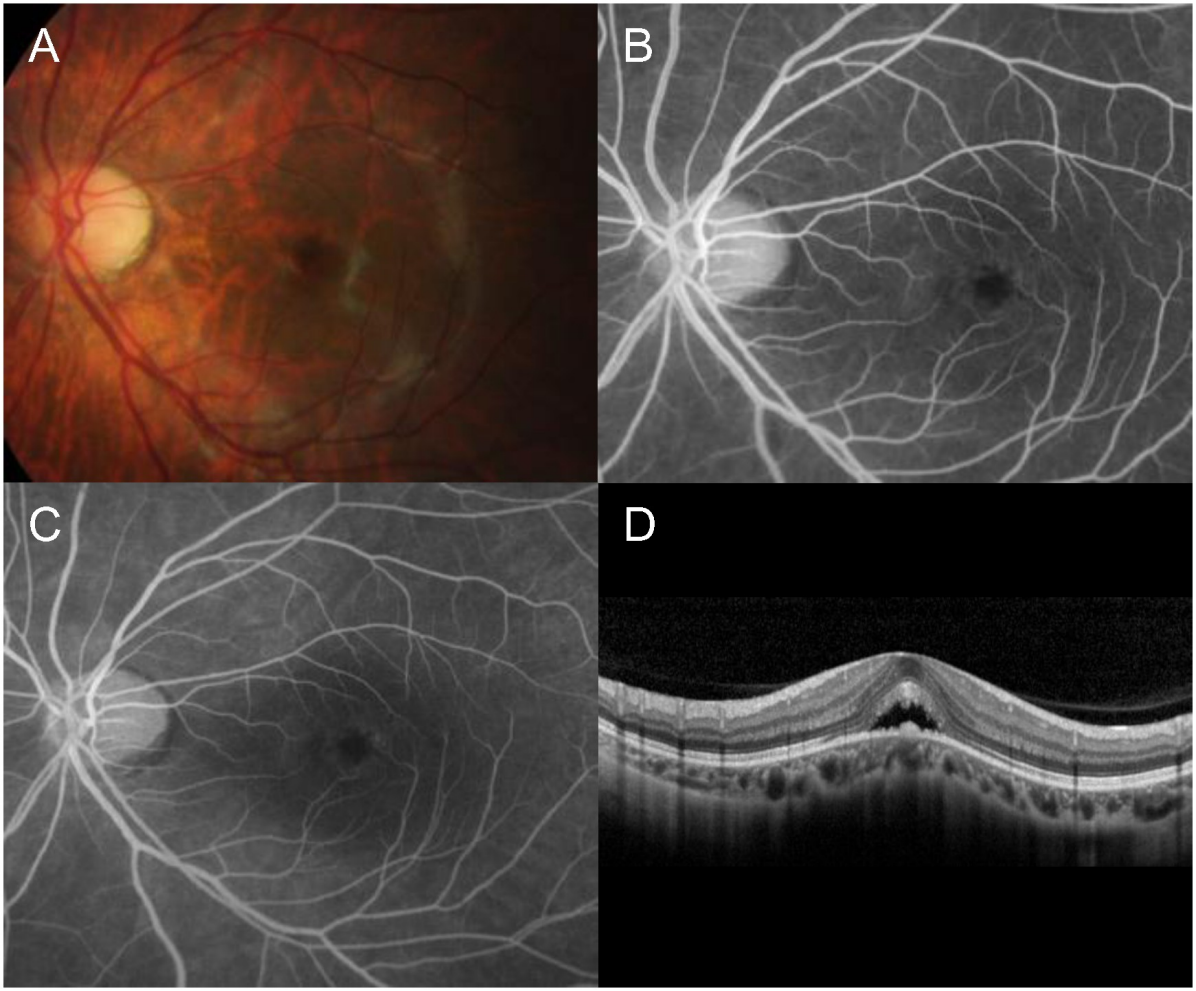
Dome-shaped macula (DSM) accompanied by serous macular detachment. (A) Fundus photography. (B) Early phase fluorescein angiography. (C) Late phase fluorescein angiography. There was no active leakage suggesting choroidal neovascularization or central serous chorioretinopathy on fluorescein angiography. (D) Optical coherence tomography images of DSM accompanied by serous macular detachment.

A total of 1,042 eyes of 615 patients was enrolled in this study. The SE was ≤−3.0 D and >−6.0 D in 679 eyes and ≤−6.0 D in 363 eyes. Tables 1 and 2 show the distribution of eyes according to age and SE. Among them, eight eyes (0.77%) of seven patients had DSM. All seven patients were Korean. Six (75%) of the eight eyes exhibited >−6.0 D of SE, and the other two eyes (25%) exhibited ≤−6.0 D of SE. The mean SE was −5.59 ± 3.61 D. All instances of DSM were noted only on vertical OCT scans. One patient was bilaterally affected, and the remaining six patients had unilateral presentations. The youngest subject with DSM was 11 years old. Detailed baseline characteristics are summarized in Table 3. In one of the eight eyes, serous detachment of the macula was noted (Fig 2). There was no evidence of choroidal neovascularization on fluorescein angiography. Retinal pathology was not observed in the remaining seven eyes. The mean height of the dome was 141.25 ± 42.71 μm, and the mean diameter of the dome base was 4,712.13 ± 571.83 μm. The outer boundary of the sclera was not identifiable in all eight eyes. None of the eyes exhibited Bruch’s membrane defect.

**Table 1:** Distribution of eyes according to age and spherical equivalents in patients with dome-shaped macula.

SE (D)	Age (years)		
	≥2 and ≤6	>6 and ≤12	>12 and ≤18
**≤−3 –>−6**	-	1 eye (12.5%)	5 eyes (62.5%)
**≤−6 –>−10**	-	-	1 eye (12.5%)
**≤−10**	-	-	1 eye (12.5%)

SE = spherical equivalent, D = Diopter

**Table 2:** Distribution of eyes according to age and spherical equivalents in patients without dome-shaped macula.

SE (D)	Age (years)		
	≥2 and ≤6	>6 and ≤12	>12 and ≤18
**≤−3 –>−6**	124 eyes (58.2%)	261 eyes (76.3%)	294 eyes (61.4%)
**≤−6 –>−10**	66 eyes (31.0%)	65 eyes (19.0%)	143 eyes (29.9%)
**≤−10**	23 eyes (10.8%)	16 eyes (4.7%)	42 eyes (8.7%)

SE = spherical equivalent, D = Diopter

**Table 3:** Baseline characteristics of patients with dome-shaped macula.

Patient	Eye No.	Age (years)	Sex	SE (diopters)	VA (logMAR)	Retinal pathologic findings
**1**	1	11	M	−3	0	-
**2**	2	16	M	−13	0.09	-
**3**	3	18	M	−3	0	-
	4	18	M	−3	0	-
**4**	5	16	F	−3.75	0	-
**5**	6	17	F	−9	0.15	-
**6**	7	17	F	−5.5	0.15	Serous macular detachment
**7**	8	14	M	−5	0	-

SE = spherical equivalent, VA = visual acuity, logMAR = logarithm of minimal angle of resolution, M = male, F = female

Table 4 shows the results of the comparison of characteristics between patients with and without DSM. Patients with DSM were significantly older (mean: 15.88 ± 2.36 years) than patients without (mean: 11.51 ± 4.60 years; p = 0.007). However, there was no difference in sex (p = 0.997), SE (p = 0.629), or BCVA (p = 0.314) between the two groups.

**Table 4:** Comparison of characteristics between patients with and without dome-shaped macula (DSM).

Characteristics	DSM	Without DSM	P-value
**N**	7 (8 eyes)	608 (1,034 eyes)	
Age (years)*	15.88 ± 2.36	11.51 ± 4.60	0.007***
Sex*	7	608	0.997**
**Male**	4	347	
**Female**	3	261	
**Spherical equivalent (diopters)**†	−5.98 ± 3.20	−5.46 ± 2.73	0.629‡
**Visual acuity (logMAR)**†	0.18 ± 0.16	0.15 ± 0.25	0.314‡

DSM = dome-shaped macula, logMAR = logarithm of minimal angle of resolution

*Comparisons between seven patients with DSM and 608 patients without it

^†^Comparisons between eight eyes with DSM and 1,034 eyes without it

^‡^Statistical analysis was performed using the Mann–Whitney *U* test

**Statistical analysis was performed using Fisher’s exact test

***Statistical analysis was performed using independent Student’s *t*-test.

## Discussion

The primary findings of the present study were as follows. First, although the incidence was very low, DSM was identified even in children and adolescents with myopia. Second, DSM was noted in relatively older subjects among our study population. Third, 75% of the eyes with DSM were not highly myopic, and there was no significant difference in SE between eyes with and without DSM.

Although almost all previously reported DSM cases were found in the adult population [1, 3–5, 7, 8, 11, 14], some were recorded in children or adolescents [10, 12, 13]. In a study by Errera et al., DSM was noted in a 12-year-old subject with congenital stationary night blindness [10]. Cebeci et al. presented a case of bilateral DSM with subretinal fluid in an eight-year-old boy without high myopia [12]. In their case, definite retinal abnormalities were noted on both fundus photography and fluorescein angiography [12].

More recently, Xu et al. reported DSM in young subjects with high myopia [13]. In their study, 17 eyes exhibited macular elevation. However, unlike typical DSM, the elevation was noted only in vertical OCT scans. Thus, this condition was considered as a distinct entity and labeled as “ridge-shaped macula.” The authors suggested that ridge-shaped macula may be due to a folding of Bruch’s membrane at the posterior pole, potentially caused by asymmetrical enlargement of that membrane in the equatorial region [13]. It is noteworthy that all the DSMs in our patients were also observed only on vertical OCT scan. Thus, it is possible that our patients were within the spectrum of ridge-shaped macula. However, there were several differences between the present study and the study by Xu et al [13]. In the latter, ridge-shaped maculae were noted in 9.2% of young subjects with myopia, an incidence that was markedly higher than that in our patients. We postulate that the primary reason for this is our inclusion of subjects with small degrees of myopia. Another difference was that two subjects were younger than 10 years in the study by Xu et al., whereas the youngest subject was 11 years old in our study, despite high myopic patients under 10 years old being included in the initial study population. We suggest that the reason for this is the paucity of DSM. Further studies with other patient cohorts are required to identify the incidence of DSM more accurately in very young subjects.

It is well-known that DSM usually develops in highly myopic eyes [1, 4, 5]. The incidence of DSM in high myopia was reported to be between 11 and 20% [1, 4, 5]. In addition, eyes with DSM generally exhibit greater degrees of myopia than those without it. In Liang et al., the mean SE values of eyes with and without DSM were −15.8 ± 3.9 D and −13.3 ± 5.0 D, respectively (p < 0.0001) [4]. However, the causal relationship of this finding—that is, whether DSM is caused by myopia or whether eyes with DSM show more severe myopia progression—has not yet been elucidated. In the present study, there was no significant difference in the degree of myopia between eyes with and without DSM. In addition, 75% of the DSM cases were found in eyes without high myopia. This result suggests that the development of DSM was not solely affected by the progression of myopia. Other factors such as an inherited trait prone to the development of DSM may have an influence. In fact, Errera et al. showed that DSM can also be found in mildly myopic or even nonmyopic eyes [10], supporting our theory. Considering that the patients with DSM were significantly older than the patients without it, this trait may be rarely expressed from a very young age if actually present.

It is not certain whether DSM is a meaningless phenomenon that occurs by chance or if it is a functional process. To provide a potential answer for this question, we focused on the previously suggested hypotheses of DSM development. Gaucher et al. postulated that a localized thickening of the choroid and resistance of the sclera to staphylomatous deformation are causes of DSM [1]. A collapse of the scleral wall caused by hypotonia in the area of the staphyloma was another potential explanation [6]. However, Imamura et al. suggested that intraocular pressure was within the normal range in all eyes with DSM, and that the scleral wall was neither inverted nor collapsed in associated OCT scans [2]. Furthermore, they suggested an adaptive mechanism to minimize defocus in highly myopic eyes as a potential cause of DSM [2]. We carefully postulate that in the distant past, compensating for myopic defocus by DSM would have been meaningful. Myopia may have been a fatal weakness to survival in the harsh environment of the prehistoric era, when survival was dependent on successful hunting and gathering. It is possible that compensation for some myopia by DSM may have helped with survival; for this reason, the trait that triggers DSM has been inherited by descendants. However, this is merely a hypothesis. Further studies are needed to identify genetic features associated with DSM and to determine whether the development of DSM compensates for myopic defocus.

Caillaux et al. classified the morphology of DSM into three types: a horizontal oval-shaped dome, a vertical oval-shaped dome, and a round dome [7]. Among these shapes, the vertical form was more frequently found than the horizontal or round one [1, 3, 4]. Coco et al. presumed that most forms of DSM could be elliptical in nature, with the longer axis located horizontally; thus, the detection of DSM would be much easier on the vertical scan with a sharper curvature [3]. In this study, DSM was noted only on vertical OCT scans, suggesting that these were likely a horizontal form of the condition. This observation may also suggest that the early stage of DSM shows morphological characteristics similar to its advanced form.

Serous detachment of the macula is a frequently observed finding in DSM. Gaucher et al. first described chronic central serous chorioretinopathy-like serous detachment without choroidal neovascularization or polypoid choroidal neovascularization [1]. Liang et al. demonstrated that the incidence of serous detachment is higher in eyes with DSM than in eyes without it [4]. However, the incidence of serous detachment markedly varied from 1.8 to 66.7% among the identified studies [1, 3, 4, 7–9, 14]. In the present study, the incidence of serous detachment was 11.1% in eyes with DSM. This incidence was similar to that reported in the adult population.

In previous studies, the mean height of the dome was between 123.2 and 429.6 μm [4, 7–9, 11, 14, 15]. The mean height of the dome in our patients was markedly smaller than previously reported values. Ellabban et al. demonstrated progressive scleral thinning in the macular regions of eyes with DSM. The scleral thinning was more pronounced in the parafoveal area than at the foveal center, resulting in an increase of the macular bulge height over time [16]. In the study by Xu et al., macular elevations were significantly lower in the ridge-shaped macular group than in the DSM group [13]. Xu et al. postulated that such gentle elevations may be characteristic for young patients [13]. Our patients were fairly young. Thus, it is possible that the measurements were largely performed at the early stage of the dome development. We believe this may be a reason for the smaller dome heights observed in our patients. Further studies are needed to identify whether the progressive increase in macular bulge height also occurs in childhood-onset DSM.

Bruch’s membrane defect is a frequently noted finding in DSM. In the study by Fang et al. [17], the incidence of Bruch’s membrane defect was 41.0% in patients with DSM. In the study by Xu et al. [13], however, Bruch’s membrane defect was noted in none of the patients with ridge-shaped macula who were <20 years old, although the incidence of the defect was 39.1% of DSM patients who were >20 years old. In the present study, Bruch’s membrane defect was not noted. In our patients, however, only cross-hair scans, which cover only a limited region of the macula, were performed, whereas the radial scans that were analyzed to detect Bruch’s membrane defect in the previous study [13] were not performed. For this reason, our finding may not confirm the absence of Bruch’s membrane defect in our patients.

Since relatively thicker sclera under the foveal region than the parafoveal region was noted in several studies [2, 18], scleral thickening has been proposed as one of the development mechanisms of DSM. However, scleral thickening may not be the only physiopathologic mechanism leading to DSM. In the study of Soudier et al. [19], choroidal thickness decreases at the periphery but not in the macular area with DSM, suggesting preserved central macular anatomy. Based on this result, Soudier et al. suggested that development of DSM may be due to macular anatomical preservation in a growing staphyloma [19]. Soudier et al. also showed increase in height of the bulge with time, resulting from perimacular extension of the myopic staphyloma [20]. Moreover, as the macular bulge increases, retinal pigment epithelium atrophy progresses [20]. In the present study with children, the height of DSM was relatively low, suggesting a less progressed form. Thus, it is possible that lack of retinal pathologic changes, such as perimacular atrophic changes or Bruch’s membrane defect, was associated with the nature of DSM in our patients. Further follow-up study will be needed to identify retinal pathologic changes with progression of myopia.

There are some limitations of this study. First, since it was a retrospective study performed in a tertiary referral center, there is a possibility of selection bias. Thus, the results of this study may not accurately reflect the general myopic population of children and adolescents. Second, although a relatively large number of patients were screened for the identification of DSM, only a few cases were diagnosed. Further studies with larger study populations are needed to elucidate the characteristics of DSM more accurately in the young population. Third, since axial length measurement was not routinely performed, SE values were used to estimate the degree of myopia. Lastly, eyes with >−3.0 diopters of SE were excluded from the study. In general, this range of myopia is considered as low-grade myopia [21]. In children, however, the definition of high myopia may differ. For instance, greater than −4.0 diopters can be considered as highly myopic when the patients are aged 5 years or younger [13]. For this reason, the exclusion criteria used in the present study may have led to selection bias. It is possible that some eyes that exhibited DSM but had smaller degrees of myopia may have been missed.

In summary, we evaluated DSM in children and adolescents with myopia. Although the incidence was less than 1%, DSM was found in very young subjects. In addition, 75% of eyes with these features were not highly myopic. These results suggest that inherent traits are involved in the development of DSM. Further long-term follow-up studies are needed to determine whether the development of DSM at a young age influences the future progression of myopia.
